# Supplementary material for: Identification of photoperiod-induced specific miRNAs in the adrenal glands of Sunite sheep (Ovis aries)
Source: Front Vet Sci. 2022 Jul 22;9:888207. doi: 10.3389/fvets.2022.888207 (PMC9354845; doi:10.3389/fvets.2022.888207)
Supplement: Supplementary file 1 [file Data_Sheet_1.ZIP › Supplementary file/miRDeep2 file.zip!/miRDeep2 file/Know_Structure/SPLP42A3/oar-mir-3957.pdf]

miRBase precursor : oar-mir-3957
Total read count : 83
oar-miR-3957-5p read count : 64
oar-miR-3957-3p read count : 19
remaining reads : 0

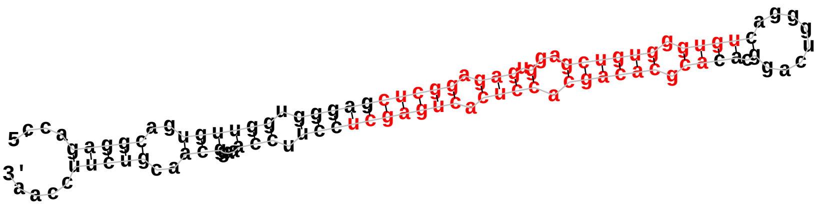

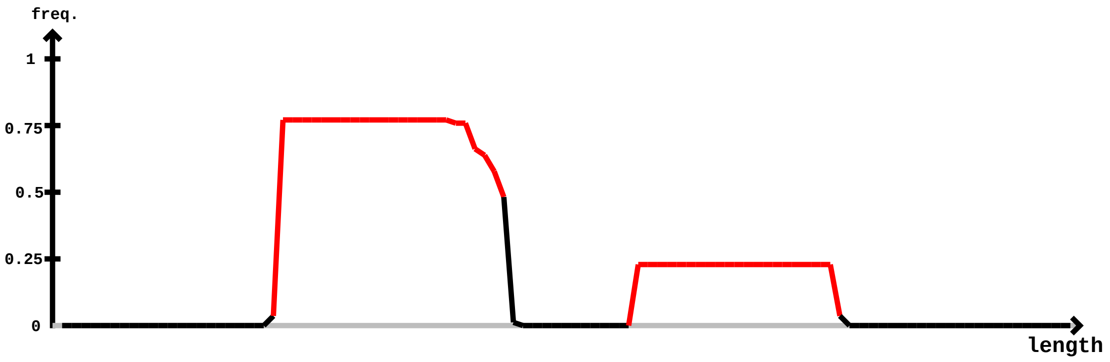


```
                     oar-miR-3957-5p
                                                          oar-miR-3957-3p
5'- ccagaggcaguguuggugggagcucggagaguggagcugugggugucagggucaggcacacgcacagcaccucacugagcuccuuccaaaggcaacgucuuccaa   -3'    exp
    ...(((((..((((((.((((((((((.(((.(..((((((.(((((........).)))).)))))).)))).)))))))))))).)))...)))..))))).... reads   mm    sample
    .....................gcucggagaguggagcugugg...............................................................   3      0     seq
    ......................cucggagaguggagcugu.................................................................   1      0     seq
    ......................cucggagaguggagcugugg...............................................................   5      0     seq
    ......................cucggagaguggagcuguggg..............................................................   2      0     seq
    ......................cucggagaguggagcugugggu.............................................................   5      0     seq
    ......................cucggagaguggagcugugggug............................................................   8      0     seq
    ......................cucggagaguggagcugugggugu...........................................................   39     0     seq
    ......................cucggagaguggagcuguggguguc..........................................................   1      0     seq
    ...........................................................acgcacagcaccucacugagc.........................   16     0     seq
    ...........................................................acgcacagcaccucacugagcu........................   3      0     seq
```
